# The cerebellum and psychiatric disorders: unraveling its role in mental health

**DOI:** 10.1055/s-0045-1810408

**Published:** 2025-08-31

**Authors:** Pedro Braga-Neto, Maria Wanessa Barbosa dos Santos, Stephanie Suzanne de Oliveira Scott, Renato Puppi Munhoz, Luiz Eduardo Novis, José Luiz Pedroso, Orlando Graziani Povoas Barsottini

**Affiliations:** 1 Universidade Federal do Ceará, Faculdade de Medicina, Departamento de Clínica Médica, Seção de Neurologia, Fortaleza CE, Brazil.; 2 Universidade do Estado do Ceará, Centro de Ciências da Saúde, Fortaleza CE, Brazil.; 3 University Health Network, Toronto Western Hospital, Morton and Gloria Shulman Movement Disorders Centre and the Edmond J. Safra Program in Parkinson's Disease, Toronto ON, Canada.; 4 Universidade Federal de São Paulo, Escola Paulista de Medicina, Departamento de Neurologia e Neurocirurgia, Unidade de Ataxia, São Paulo SP, Brazil.

**Keywords:** Cerebellum, Mental Disorders, Mental Health

## Abstract

Several studies describe a strong association between structural and functional abnormalities of the cerebellum and psychiatric disorders. It is possible to find investigations especially in cases of schizophrenia, bipolar disorder, depression, anxiety disorders, attention-deficit/hyperactivity disorder (ADHD), and autism spectrum disorder (ASD). The involvement of the cerebellum in these conditions is also supported by clinical, functional, and structural imaging studies. The present narrative review aims to discuss and highlight the role of the cerebellum in these disorders, gathering evidence on the possible locations and connections of the affected cerebellar areas and their implications in the cognitive, emotional, and behavioral domains.

## INTRODUCTION

The cerebellum is traditionally known for its role in motor-related functions. However, increasing evidence suggests that the cerebellum plays a much broader role, including the modulation of social and affective behaviors. Current research indicates the cerebellum's influence on subcortical regions, as well as on cortical processes that extend beyond the motor domain. 1 2 Notably, several studies have found strong associations between structural and functional abnormalities of the cerebellum and various psychiatric disorders, particularly schizophrenia, bipolar disorder, depression, anxiety disorders, attention-deficit hyperactivity disorder (ADHD), and autism spectrum disorder (ASD). The involvement of the cerebellum in these conditions is further supported by clinical, functional and structural imaging studies. 3

The present narrative review aims to comprehensively discuss and highlight the role of the cerebellum in various psychiatric disorders, in addition to endorsing the need for the development of studies that deepen the understanding of this complex and functionally-diverse structure. Table 1 systematically gathers the main anatomical regions of the cerebellum associated with different psychiatric disorders. The selection of these areas is based on previous studies focused on new discoveries about cerebellar functioning. Table 1 aims to point out the most recurrent neuroanatomical and psychiatric correlations, serving as a complementary resource that organizes, in a concise manner, the content that will be further explored throughout the subsections of the present review.

**Table 1:** Cerebellum's anatomical areas associated with psychiatric disorders

Psychiatric disorders	Cerebellum's anatomical areas
Schizophrenia	Lobules IV, V, VII, and VIII, and Crus I–II
Autism	Vermis and cerebellum–ventral tegmental area
Attention deficit hyperactivity disorder	Posterior inferior cerebellar vermis and posterior inferior lobules
Depression	Posterior cerebellum and vermis
Bipolar disorder	Right hemisphere, including lobules I–IV, V and Crus I and II
Anxiety	Posterior vermis and amygdala

**Figure 1:**
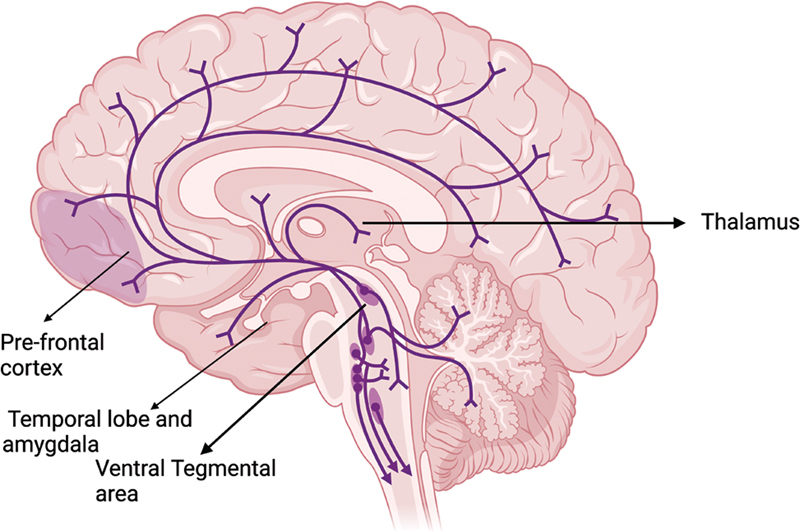
Main brain areas with connections to the cerebellum associated with psychiatric disorders.

Figure 1 is another visual resource that aims to provide a simplified understanding of the main neural communication pathways between the cerebellum and important brain structures. In this graphic representation, a complex network of connections can be observed extending from the cerebellum to important regions such as the prefrontal cortex, temporal lobe, amygdala, ventral tegmental area, and thalamus. These cerebellar connections point to a role different from the traditionally recognized motor functions, highlighting its participation in cognitive and emotional processes.

## THE ROLE OF THE CEREBELLUM IN MENTAL HEALTH: ANATOMICAL AND PHYSIOLOGICAL BASES

The study of cerebellar anatomy dates to the 18th century, specifically to 1776, when Vincenzo Malacarne quantified the internal structures of the cerebellum, numbering its laminae and correlating them with intellectual faculties. Malacarne continued to make significant contributions, progressing from neuroanatomy to neurophysiology in his work on understanding the cerebellum. 4

The cerebellum is a complex structure that works in tandem with the cerebral cortex and basal ganglia as a movement co-processor. 5 In humans, 60 to 80% of the brain's neurons are located in the cerebellum, a proportion that underscores its immense potential and capacity. 6 This makes the cerebellum a crucial component in information processing and the planning of sensory-motor activities. It is involved in a wide range of both motor and non-motor functions, thanks to its intricate circuits and extensive connectivity with other areas of the brain. 7

This neural structure is strongly connected to the cerebral hemispheres through a network of afferent and efferent pathways. The afferent pathways include the corticopontine and pontocerebellar tracts, while the efferent pathways are the cerebrothalamic and thalamocortical tracts. 7 The modulation of the cerebellum to higher functions is supported by the corticopontocerebellar pathways, such as the temporopontine connections. The superior temporal lobe is associated with higher order behavior probably due to its anatomical interconnections with the parietal and frontal association areas and the paralimbic regions of the inferotemporal, cingulate, and orbital frontal cortices. That area is also connected to the pons and the cerebellum by the corticopontocerebellar system, reiterating the role of the cerebellum in modulating behavior and cognition. 8 9

The cerebellum is traditionally divided into three lobes: the anterior lobe, the posterior lobe, and the flocculonodular lobe. However, Larsell classified the cerebellum into 10 lobes, with the anterior lobe consisting of lobes I to V, the posterior lobe made up of lobes VI to IX, and the flocculonodular lobe as lobe X. Lobule VII is further subdivided into crus I, II, and VIIB in the hemispheres. 10 Each region of the cerebellum plays a role in coordinating movement planning, and lesions in these regions can lead to specific ataxic symptoms. Common symptoms of ataxic disorders include abnormalities in ocular mobility, limb incoordination, impaired gait and balance, tremors, and speech difficulties. 11

For nearly 200 years, research on the cerebellum has primarily focused on its role in motor function, as well as the movement and coordination impairments that result from alterations in this structure. 12 While its involvement in motor functioning is well-established, the cerebellum is also connected to regions associated with various non-motor functions. Recent studies emphasize that the cerebellum plays a significant role in processes related to perception, attention, emotion, and cognition. 13 14 15

Clinical and radiological studies reveal that the anterior portion of the cerebellum is involved in motor control, the flocculonodular lobe plays a role in affective processing and vestibulocerebellar functions, and the posterior portion is responsible for complex cognitive operations. 16 Deficits associated with cerebellar damage can be categorized into four main areas: impairments in executive functions, visuospatial cognition, linguistic abilities, and personality changes. Alterations in the posterior region of the cerebellum can lead to executive function deficits similar to those observed in prefrontal cortex injuries. These deficits may include difficulties with planning, task organization, action sequencing, verbal fluency, working memory, abstract reasoning, problem-solving strategies, task switching, and managing multiple activities simultaneously. 17

Lesions in the cerebellum can lead to significant impairments in visuospatial processing and linguistic abilities. Linguistic deficits may include agrammatism, changes in speech intonation (dysprosody), difficulty in sentence structuring (syntax), and reduced verbal fluency. When lesions affect the vermal and paravermal regions of the cerebellum, socially inappropriate behaviors, mood disturbances, disinhibition, and regressive or compulsive behaviors may emerge. From a neurobehavioral standpoint, these deficits impact emotional regulation and social interactions and are often associated with symptoms of ASD and psychotic disorders. 17

The role of the cerebellum and its cerebrocerebellar connections is well established. With the advent of functional magnetic resonance imaging (fMRI) and positron-emission tomography (PET), a detailed functional map of the cerebellum has become possible. 18 This mapping has enhanced our understanding of the cerebellum's complex functions and has led to new insights into neurological and psychiatric disorders, including the cerebellar cognitive-affective syndrome (CCAS), which is characterized by deficits in executive functions, linguistic processing, spatial cognition, and affect regulation in patients with cerebellar disease, mood disorders, ASD, and ADHD. 6 19 Figure 1 illustrates the main brain areas with connections to the cerebellum that are associated with these psychiatric disorders.

## THE ROLE OF THE CEREBELLUM IN PSYCHOSIS

Psychosis is a complex neuropsychiatric syndrome, representing a combination of symptoms rather than a diagnosis. These symptoms or signs include, but are not restricted to, hallucinations, delusions, disorganized thoughts and motor behavior. From the motor perspective, it may include agitation, restlessness, random not-purposeful movements, and lack of responsiveness. 20 Psychosis, therefore, can occur as part of diverse conditions, such as schizophrenia, bipolar disorder, major depression (with psychotic features), substance abuse disorders, infections, and various forms of brain diseases. 20

From an anatomical and functional perspective, brain imaging studies have found abnormalities in different regions, including frontal and temporal regions atrophy, and abnormal connectivity among the prefrontal regions, thalamic nuclei, and cerebellum. These later circuit disruption findings led to the designation *cognitive dysmetria* , encompassing difficulty in prioritizing, analyzing, organizing, and responding to information. Importantly, these changes affect micro and macro structure, chemical transmission and connectivity, development, and plasticity. As such, the cerebellum, classically part of brain structures with roles in movement coordination and control, also has functions related to cognition and emotion, also known as CCAS. 21 22

### The cerebellum and schizophrenia

Recent advances in studies of the cerebellum regarding schizophrenia have emerged in the past decades. The cerebellum plays a key role in many aspects of cognitive processing and is both functionally and anatomically connected to brain regions critical to cognitive and emotional processing, such as the prefrontal cortex, basal ganglia, and ventral tegmental area. 21 22 Additionally, the cerebellum per se has also shown to present with multiple abnormalities in this context:

- Reduced cerebellar volume in both gray and white matter thickness affecting lobules IV, V, VII, and crus I, II, and VIII ( Table 1 );
- Microcircuit and cellular changes, including reduced density of granule and Purkinje cells, and inhibitory interneurons, and altered reduced branching and synaptic connectivity;
- Decreased functional activation of specific cerebellar areas during cognitive tasks (cerebellar lobules IV, V, VI, crus I and II, VIIB, VIIIA, and VIIB);
- Neurotransmitter dysregulation, including dopaminergic, serotonergic, glutamatergic, and GABAergic systems. 21 23 24

Moreover, these connectivity alterations are bidirectional, suggesting that, in the context of schizophrenia, they could be the result of neurodevelopmental disruptions or compensatory mechanisms, both influenced by abnormalities in neurotransmitter systems. 21 23

### The cerebellum and cognitive dysmetria

It is no surprise that the cerebellum is an essential player in the cognitive dysmetria model of psychosis, in which impaired coordination of mental processes (attention, memory, motor activity) underlies diverse psychotic symptoms. Accordingly, studies showed that cerebellar abnormalities are predictors of psychotic symptoms such as sensorimotor deficits, cognitive dysfunction, and hallucinations. A classic symptom in schizophrenia, hallucinations are linked to altered sensory feedback processing, potentially involving cerebellar dysfunction in motor action predictions, given its complex connections with cortical areas. However, the specific role of the cerebellum in positive symptoms of schizophrenia remains unexplored, with limited findings in meta-analyses of structural MRI studies. 21 25

### The cerebellum and predictive processing

Dysfunction in the predictive signals that facilitate learning may contribute to psychosis. Physiologically, neural systems are able to predict patterns in the environment based on past experiences. 23 26 These predictions reduce discrepancies between expectations and actual outcomes in a process that requires continuous updating, leading to a dynamic shaped internal model of the external world. As such, disruptions in predictive coding may contribute to wrong interpretations of perceptions and beliefs that are part of hallucinations and delusions. 26 27 Additionally, regarding these positive signs seen in psychotic patients, abnormal prediction-based attenuation of sensory processing could potentially account for the heightened sensory cortex activity observed in studies of psychotic symptoms. 23 26 27

In conclusion, several positive and negative symptoms that are main contributors to disability in the context of psychosis are still suboptimally managed by current therapies. The investigation of cerebellar involvement in psychosis is a promising unexplored venue for therapeutic intervention.

## THE CEREBELLUM AND AUTISM

Autism, or autism spectrum disorder (ASD), is a neurodevelopmental disorder characterized by stereotyped patterns of interests, activities, and behavior, with difficulties in social interactions and verbal communication. In recent years the relation between cerebellum and ASD has been extensively studied, with many reports of histological and morphological abnormalities of the cerebellum related to different autistic phenotypes. 28 The dysfunction of closed-loop cerebellar circuits with cerebral cortical regions may impact on the core ASD symptoms of social and communication deficits and repetitive and stereotyped behaviors. 19

Zhou et al. suggested that cerebello-ventral tegmental ( Table 1 ) area circuit dysfunction may contribute to ASD pathogenesis and autistic phenotypes, including the presence of stereotyped behavior, hypohedonia, and impaired social interaction. 29 Wang et al. studied brain images from 817 children (age 5–18), using structural T1-weighted brain MRIs. They observed differences in cerebellar areas between individuals with and without autism, particularly involving parahippocampal gyrus, pars opercularis, and transverse temporal gyrus in the right hemisphere. 30

Yenkoyan et al., using genetic rodent models of ASD, suggested that genes related to structural and functional alterations in the cerebellum, including *Fmr1* , *Mecp2* , *Tsc1/2* , and *Nlgn3/4* mutations, may be linked to ASD. 31 Genetic analysis demonstrated that some genes implicated in ASD, like *SHANK3* , *EN2* , *RORA* , are also involved in cerebellar development. Heterozygous mutations of the *CHD8* gene are associated with a high penetrance of autism and other neurodevelopmental phenotypes. *CHD8* is expressed in the developing central nervous system at all stages of development, with moderate expression in the cerebellum, hippocampus, olfactory bulb, and also in the neocortex. 32

Other studies demonstrate an extensive pathology in the cerebellar areas of patients with autism, including vermal hypoplasia, gray and white matter involvement, and Purkinje cells loss. In addition to structural changes, oxidative stress, inflammation, apoptosis, and GABAergic function are altered in the cerebellum of patients with ASD. 33

## THE CEREBELLUM AND ATTENTION DEFICIT HYPERACTIVITY DISORDER

Attention deficit hyperactivity disorder) is a neurodevelopmental disorder characterized by inattention, hyperactivity, and impulsivity that are incompatible with the individual's developmental stage and tend to cause impairments. The most common forms of ADHD have predominant symptoms of inattention, hyperactivity, or a combination of both. The manifestations of the disorder vary and will depend on the individual's specific characteristics. Attention deficit hyperactivity disorder is observed in 5 to 10% of school-aged children. It is a disorder that persists throughout an individual's life and affects both females and males, impacting 2 to 6% of the global population. 1 2

Despite the association of this disorder with a global reduction in brain volume, specific alterations were observed in MRI, such as a decrease in gray matter in areas located in the frontostriatal circuits. An altered volume in the white matter was also identified in different neural tracts, which points to possible impairments in communication in some of these participating regions. Among the identified changes is cortical thinning, and some studies indicate that cortical development may be slower in children with the disorder, who only reach the expected level of cortical thickness three years later than children without the disorder. 34

Considering this, ADHD is associated with complex structural changes involving various brain regions and their connections, with the main ones being the prefrontal cortex, cerebellum, basal ganglia, white matter, thalamus, and amygdala. More specifically, regarding the cerebellum, the primary area affected is the posterior inferior cerebellar vermis ( Table 1 ). 34 35 No reduction in the posterior vermis was found in children treated with methylphenidate, the most commonly used medication to treat ADHD. Significant structural changes were observed in adults with the disorder who had not undergone medication intervention. 36

Many neuropsychological models have attempted to explain the behavioral and cognitive manifestations of ADHD. Among the main ones are deficits in executive function (EF) and temporal processing. Executive function is used to describe higher-order cognitive functions, including inhibitory control and working memory. Deficits in EF have been shown in neuropsychological models of ADHD and converge with structural changes observed in the brain's frontal regions. From a behavioral perspective, deficits in EF may lead to forgetfulness, difficulty planning, and coordinating everyday tasks. Other theories add delay aversion, based on evidence that children with ADHD show a preference for smaller immediate rewards rather than larger delayed ones. They also exhibit deficits in temporal processing, which makes children tend to overestimate the passage of time. This may explain the difficulty in waiting one's turn. 34

Giedd et al.'s study used quantitative MRI to examine brain differences in 112 boys with ADHD in comparison to healthy controls. They identified that the volumes of the cerebellar hemispheres were smaller in boys diagnosed with ADHD. In a follow-up study with the same sample, it was found that both the cerebellar vermis and the posterior-inferior lobules were significantly smaller in individuals with ADHD ( Table 1 ). This could indicate that dysfunction in the cerebello-thalamo-prefrontal circuit may be associated with deficits in motor control, inhibition, and executive functions shown in ADHD. 37 38

Structural differences in the cerebellum are among the most relevant pieces of evidence in ADHD. A reduction in the total volume of the cerebellum and the superior vermis has been found, which persists throughout the development of individuals with ADHD. A decrease in the right cerebellum, located in the posterior vermis and the posterior lobes of the cerebellum, is also observed. Additionally, crus I and lobule IX have been linked to the symptomatology present in the disorder. The severity of ADHD symptoms has been associated with the degree of cerebellar vermis reduction, as well as with the total decrease in cerebellum size. A reduction in the volume of the posterior cerebellum has also been associated with more severe symptoms. 39

Altered patterns of cerebellar connectivity have been found in patients diagnosed with ADHD, and reduced connectivity with the prefrontal cortex has been identified. When observing striatal connectivity in a large sample of individuals with ADHD and comparing it to their typical relatives, a stronger correlation was observed between symptoms of hyperactivity, impulsivity, and inattention and increased connectivity of the posterior putamen with the cerebellum and occipital cortex. 40 41

## THE CEREBELLUM IN AFFECTIVE DISORDERS

Approximately 30 years ago, the description of cognitive and affective symptoms in patients with cerebellar disease was published by Schmahmann et al., and it was named CCAS. Evidence is increasingly emerging correlating cerebellar disease with affective symptoms, such as depression, personality changes characterized by blunted affect, and inappropriate or disinhibited behavior. 42

It is believed that the “emotional cerebellum” includes several specific and non-specific areas, and each emotion would recruit specific cerebellar loci. Data supports that there is strong posterior vermal and paravermal cerebellar activation when it comes to primary emotions, like happiness, sadness, anger, fear, or disgust. 43

Based on the connections between the posterior vermis and the limbic structures of the brain, this area was called the “limbic cerebellum,” because it is involved in the modulation of emotional process. The anatomical substrate for the influence of the cerebellum on affective/limbic and autonomic behavior is the connectivity of the cerebellar vermis and fastigial nucleus, with subcortical limbic, associative, and paralimbic cortical areas. Studies done with functional MRI described that when viewing emotional versus neutral pictures, regions of the cerebellum including a cluster in the crus II area extending to the midline were activated, and that could be important for the affective aspects of the CCAS. 16 44

### The cerebellum and depression

Depression is a common comorbidity when it comes to chronic illnesses; however, the prevalence of depression in patients with degenerative cerebellar disorders is unusually high. 45 Structural imaging studies have shown that major depressive disorder and higher depressive symptoms are related with smaller gray matter volume in the total cerebellum as well as cerebellar subregions, including the posterior cerebellum and vermis ( Table 1 ). Functional studies show that abnormal cerebellar-cerebral resting-state connectivity can discriminate patients with depression from healthy controls. Interestingly, patients that undergo treatment for depression have their cerebellar connectivity to the amygdala and hypothalamus normalized after treatment. 43

### The cerebellum and bipolar disorder

Bipolar disease (BD) is a disorder characterized by mood that cycles between depression and mania. Anatomical and functional studies have reported that the cerebellum has a role in the pathophysiology of the disease. When compared with healthy controls, BD patients have a pattern of reduced gray matter density in specific cerebellar regions, particularly in the right hemisphere, including lobules I to IV, V, and crus I and II ( Table 1 ), suggesting that these alterations may contribute to mood dysregulation and cognitive dysfunction in bipolar disorders, perhaps encompassing motor and emotional symptoms like impulsivity, disinhibition and hyperactivity. 46 Studies suggest that there are anomalies in cerebellar metabolism of gamma-aminobutyric acid (GABA) synthesizing protein, and in the metabolite N-acetylaspartate and choline ratio in patients with BD, contributing to the pathophysiology of the disease. 43

### The cerebellum and anxiety

Anxiety disorders are among the most prevalent psychiatric conditions worldwide and are characterized by recurrent worrying thoughts and tension, notably when the source is uncertain. Changes in function and structure of the cerebellum have been associated with social anxiety disorders. Studies correlated anxiety disorders with abnormalities in the cerebellar posterior vermis and increased connectivity between the cerebellum and the amygdala and the posterior cingulate ( Table 1 ). Studies have shown that there is an increase in gray matter volume on the left cerebellum of patients with social anxiety and that, upon treatment with selective-serotonin reuptake inhibitors, there was a decrease in the volume of the left cerebellum and a reduction of the anxiety symptoms. 13

### Affective symptoms in different etiologies

Symptoms of affect have been reported with increasing frequency in patients with cerebellar ataxia. A study published by Maas et al. in 2021 applied the CCAS scale to 20 patients with SCA 3, with mild-to-moderate ataxic symptoms, and detected cognitive and affective symptoms in these patients at an early stage of the disease. It was also reported that affective symptoms were strongly correlated with the degree of cognitive impairment but had no correlation with motor impairment. 47

Case reports from cerebellar stroke patients have provided details about the personal impact of the disease on affective symptoms. Patients presented emotional dysregulation and aggression, blunted affect, agitation, temporal and spatial confusion, personality changes with dysphoria, disinhibition, affective indifference toward close family members and panic disorders. 44

A study with 300 patients with SCA 1, 2, 3, and 6 evaluated these patients every 6 months for a period of 2 years and demonstrated that up to 26% of patients had depression. However, the prevalence of suicidal thoughts is considerably higher in patients with SCA 3 when compared with other SCAs, even with a similar ataxia severity score between the groups, suggesting that depression in patients with SCA 3 may be the result of a neurodegenerative process, not just an affective response to a motor limitation. 48

According to Hoche et al., children with ataxia-telangiectasia presented progressive affective symptoms over the years. They evaluated 20 patients, divided into groups (preschool, school, and adolescents/young adults), to whom the children's version of the reading the mind in the eyes test (RMET-C) was applied, which is a social cognition test. When compared with controls, patients showed deficits in emotion recognition, particularly for positive emotions, and affect dysregulation was noticed in some patients. 49

In Friedreich ataxia (FRDA), depression plays an important role in the health and wellbeing of patients. Some studies have specifically investigated neuropsychiatric symptoms, such as depression, anxiety, and psychotic symptoms in this group of patients. A study performed in Spain with 57 patients with FRDA, in which a depression questionnaire was administered, concluded that patients with the disease had more depressive symptoms when compared with the general population, specifically complaints such as sadness, pessimism, loss of pleasure, suicidal thoughts, feelings of worthlessness, loss of energy, and irritability. 50 Another study with patients with FRDA in the Czech Republic evaluated 33 patients with the Mild Behavioral Impairment Checklist (MBI-C), a new instrument that assesses neuropsychiatric symptoms in patients with neurodegenerative diseases, which encompasses five domains: decreased motivation, emotional dysregulation, lack of impulse control, inappropriate social behavior, and abnormal perception of thought content, and demonstrated that depressive and anxious symptoms, as well as a decrease in motivation, are present in these patients. 51

## CLINICAL AND THERAPEUTIC IMPLICATIONS OF THE CEREBELLUM IN PSYCHIATRIC DISORDERS

Current treatment strategies for cerebellar dysfunction in psychiatric disorders predominantly involve non-invasive brain stimulation (NIBS) techniques and cognitive rehabilitation. Transcranial magnetic stimulation (TMS) and transcranial direct-current stimulation (tDCS) are the most utilized NIBS methods. These techniques target the cerebellar vermis and have shown promise in alleviating negative and depressive symptoms in schizophrenia while enhancing frontal-cerebellar connectivity. They are generally well tolerated, with mild and transient side effects being the most frequently reported. 52 53 However, larger-scale studies are necessary to fully assess the clinical efficacy of these techniques in this context.

Cognitive rehabilitation programs are also used to address the cognitive deficits associated with cerebellar dysfunction, focusing on improving executive function, attention, and working memory. These programs can be tailored to meet the individual needs of patients with cerebellar abnormalities. 54 Pharmacotherapy targeting neurotransmitter systems involved in cerebellar function, such as GABAergic and glutamatergic pathways, is another promising area of investigation. Although specific pharmacological treatments for cerebellar dysfunction remain underexplored, medications that modulate these neurotransmitter systems may offer therapeutic benefits. However, further research is needed to identify effective pharmacological interventions. 55

Looking ahead, future treatments for cerebellar dysfunction in psychiatric disorders are likely to involve advanced neuromodulation techniques, biomarker development, and gene therapy. Optimizing non-invasive brain stimulation (NIBS) parameters alongside neuroimaging could enhance our understanding of the cerebellum's role in psychiatric conditions and facilitate more targeted interventions. 54 The development of biomarkers is essential for early diagnosis and personalized treatment, as well as for identifying neurophysiological markers of cerebellar dysfunction, which could guide the application of neuromodulation techniques and monitor treatment outcomes. 56 Although still experimental, gene therapy and stem cell approaches show promise for addressing cerebellar dysfunction at a molecular level, offering new treatment possibilities in the future. 56

In conclusion, growing evidence and recent data suggest that the cerebellum plays a role in several psychiatric disorders. A limitation on the current research is that clinical, pathological, and neuroimaging studies support its involvement, but the majority of research remains inconclusive when it comes to identifying specific anatomical abnormalities in the cerebellum associated with these disorders. More studies should be performed to create causal evidence of the involvement of the cerebellum in neuropsychiatric disorders and, perhaps, with the more promising neuromodulation strategy as a treatment that could focus on the cerebellum, there will be more evidence of its involvement facilitating possible future treatments.
